# Retraction Note: *HNF4A-AS1*/hnRNPU/CTCF axis as a therapeutic target for aerobic glycolysis and neuroblastoma progression

**DOI:** 10.1186/s13045-023-01493-7

**Published:** 2023-08-15

**Authors:** Huajie Song, Dan Li, Xiaojing Wang, Erhu Fang, Feng Yang, Anpei Hu, Jianqun Wang, Yanhua Guo, Yang Liu, Hongjun Li, Yajun Chen, Kai Huang, Liduan Zheng, Qiangsong Tong

**Affiliations:** 1 Department of Pediatric Surgery, Union Hospital, Tongji Medical College, Huazhong University of Science and Technology, 1277 Jiefang Avenue, Wuhan, 430022 Hubei Province People’s Republic of China; 2 Clinical Center of Human Genomic Research, Union Hospital, Tongji Medical College, Huazhong University of Science and Technology, 1277 Jiefang Avenue, Wuhan, 430022 Hubei Province People’s Republic of China; 3 Department of Pathology, Union Hospital, Tongji Medical College, Huazhong University of Science and Technology, 1277 Jiefang Avenue, Wuhan, 430022 Hubei Province People’s Republic of China

**Retraction Note: Journal of Hematology & Oncology (2020) 13:24** 10.1186/s13045-020-00857-7

The Editor-in-Chief has retracted this article. After publication, concerns were raised about image reuse in the article. Specifically:

The top-left panel of Fig. 2F was reproduced in two panels of Fig. 4H, and in Fig. S11E of the supplementary materials.

There is overlap between the top-left panel of Fig. 2G and the second-from-top-right panel of Fig. 4I.

There is overlap between the top-left panel of Fig. 4I and the bottom-left panel of Fig. 5G.

There is overlap between the second-from-bottom-left panel of Fig. 4I and the top-right panel of Fig. 5G.

There is overlap between the bottom middle panel of Fig. 5G and the bottom-right panel of Fig. S7B of the supplementary materials.

Additionally, the top-left panel of Fig. 2F was found to have previously been published in [1] by some of the same authors. The Editor-in-Chief no longer has confidence in the data reported in the article.

Qiangsong Tong has stated on behalf of all authors that they do not agree to this retraction.
